# Floristic Diversity of Riparian and Associated Vegetation Along the Amnay River (The Philippines)

**DOI:** 10.1002/pei3.70122

**Published:** 2026-02-04

**Authors:** Enrico L. Replan

**Affiliations:** ^1^ Institute of Environmental Science and Meteorology, College of Science University of the Philippines Diliman Quezon City Philippines

**Keywords:** Amnay River, floristic diversity, Mindoro, restoration, riparian vegetation, riverbank ecology

## Abstract

Riparian vegetation plays a critical role in maintaining ecological integrity along river corridors, yet it is highly sensitive to changes in hydrological and geomorphic conditions, particularly in sediment‐influenced tropical river systems. In many Philippine rivers, increased sediment deposition and landscape disturbance have altered riparian structure and floristic composition, underscoring the need for site‐specific baseline assessments. This study aimed to document the floristic composition, vegetation structure, and spatial patterns of riparian vegetation along the downstream reach of the Amnay River in Occidental Mindoro, Philippines, as a baseline for understanding current riparian conditions in a sediment‐influenced river corridor. Vegetation surveys were conducted using transects and quadrats established along the riverbanks, where species composition, growth form, and structural attributes were recorded. Species importance values and diversity indices were calculated to characterize vegetation dominance and diversity patterns across sampling sites. Riparian vegetation communities were mapped to describe spatial distribution and fragmentation. A total of 125 plant species representing 40 families were recorded, with vegetation dominated by herbaceous and disturbance‐tolerant taxa, particularly members of Poaceae and Fabaceae. Woody vegetation was limited and occurred mainly as isolated remnant patches, resulting in low vertical complexity and fragmented spatial structure. Species diversity varied among sampling sites, reflecting localized differences in substrate stability and vegetation cover. The findings provide a baseline characterization of riparian vegetation in a sediment‐influenced tropical river system and highlight the persistence of remnant woody vegetation within an otherwise simplified riparian corridor. This baseline information is essential for future monitoring, comparative studies, and the evaluation of riparian management and restoration efforts.

## 1 Introduction

Riparian ecosystems provide essential ecological functions, including erosion control, sediment retention, flood mitigation, and support for biodiversity (Naiman and Décamps 1997). Riparian vegetation enhances these functions by stabilizing riverbanks, regulating microclimate, filtering sediments and pollutants, and providing habitat for a wide range of organisms (Tabacchi et al. 2000). In tropical regions, riparian formations often harbor distinct assemblages of native flora that serve as ecological buffers within agricultural and human‐modified landscapes (Posa et al. 2008; Bhatta et al. 2022). These riparian formations occur within broader Philippine Forest types that range from grassland–shrubland mosaics to lowland tropical forest formations (Fernando et al. 2008).

Despite their ecological importance, riparian systems worldwide are among the most heavily altered ecosystems. Agricultural expansion, settlement development, reclamation, quarrying activities, and natural sedimentation processes have resulted in extensive siltation and altered hydrological regimes (Ekka et al. 2020). These pressures frequently exceed the capacity of rivers to maintain stable riparian corridors, leading to habitat degradation and fragmentation (Naiman et al. 2005). In the Philippines, riverbank conversion and agricultural intensification have accelerated sediment deposition, resulting in widespread habitat loss and the fragmentation or complete removal of riparian vegetation (Malabrigo Jr. et al. 2014). In such contexts, remaining vegetation patches are often too small and isolated to sustain diverse plant communities or support effective regeneration (Sarmiento et al. 2022).

The Amnay River, part of the Amnay–Patrick Watershed Basin, is the longest river system in Barangay Claudio Salgado, Municipality of Sablayan, Occidental Mindoro, with its headwaters originating from Mt. Iglit–Baco National Park. The river supports remnant vegetation formations ranging from grasslands and shrublands to open forest. However, substantial sediment and sand discharge from upstream erosion has compromised channel conveyance capacity, particularly in the midstream and downstream sections. These conditions have been exacerbated by agricultural expansion, river sand quarrying, and reclamation activities along the downstream reaches.

As a result, downstream sections of the Amnay River have experienced extensive sediment accumulation, sandbar formation, and channel migration, leading to the burial and partial removal of riparian vegetation. Analysis of multi‐temporal satellite imagery from 1997 to the present indicates a progressive decline and spatial fragmentation of vegetation cover, driven by recurrent sedimentation and siltation. These processes have altered substrate conditions, reduced plant survival and regeneration, and constrained the persistence of woody riparian vegetation.

Given the current physical condition and altered ecological state of the downstream reaches, comprehensive floristic surveys have not previously been conducted, resulting in limited baseline information on plant species composition and distribution. Consequently, no distinct plant assemblages have been formally associated with the river system. In this context, the present study provides a baseline account of existing riparian vegetation along the downstream section of the Amnay River. Specifically, it aims to document species composition and habitat associations, identify dominant and threatened taxa, and establish an initial reference for assessing riparian ecological condition and recovery potential. By characterizing the remaining vegetation under prevailing siltation conditions, this study contributes foundational information to support future river restoration and management efforts focused on improving river ecosystem health (Zeng et al. 2019).

## 2 Materials and Methods

### 2.1 Study Area

**FIGURE 1:**
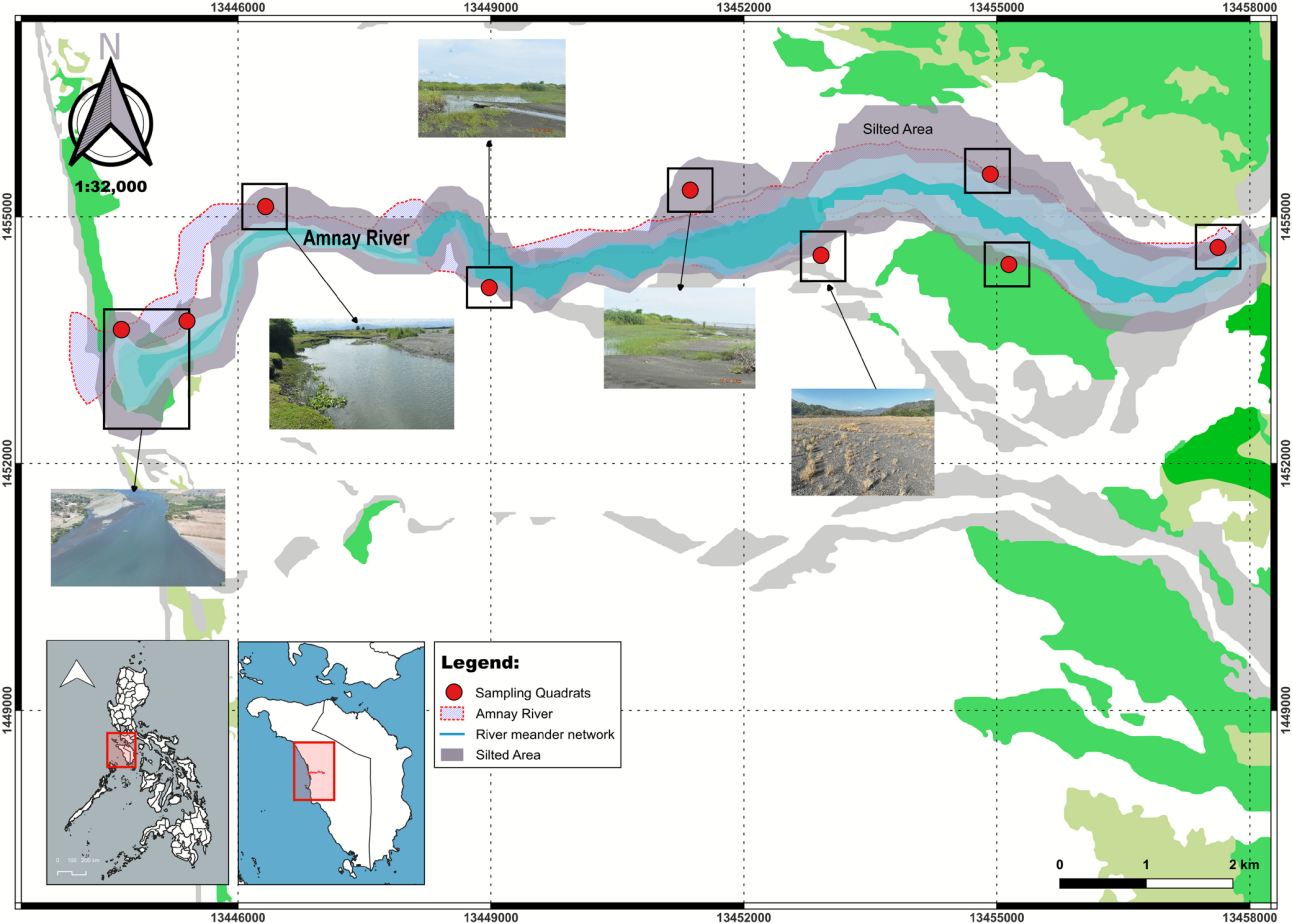
The location map of the study area and the sampling points.

**FIGURE 2:**
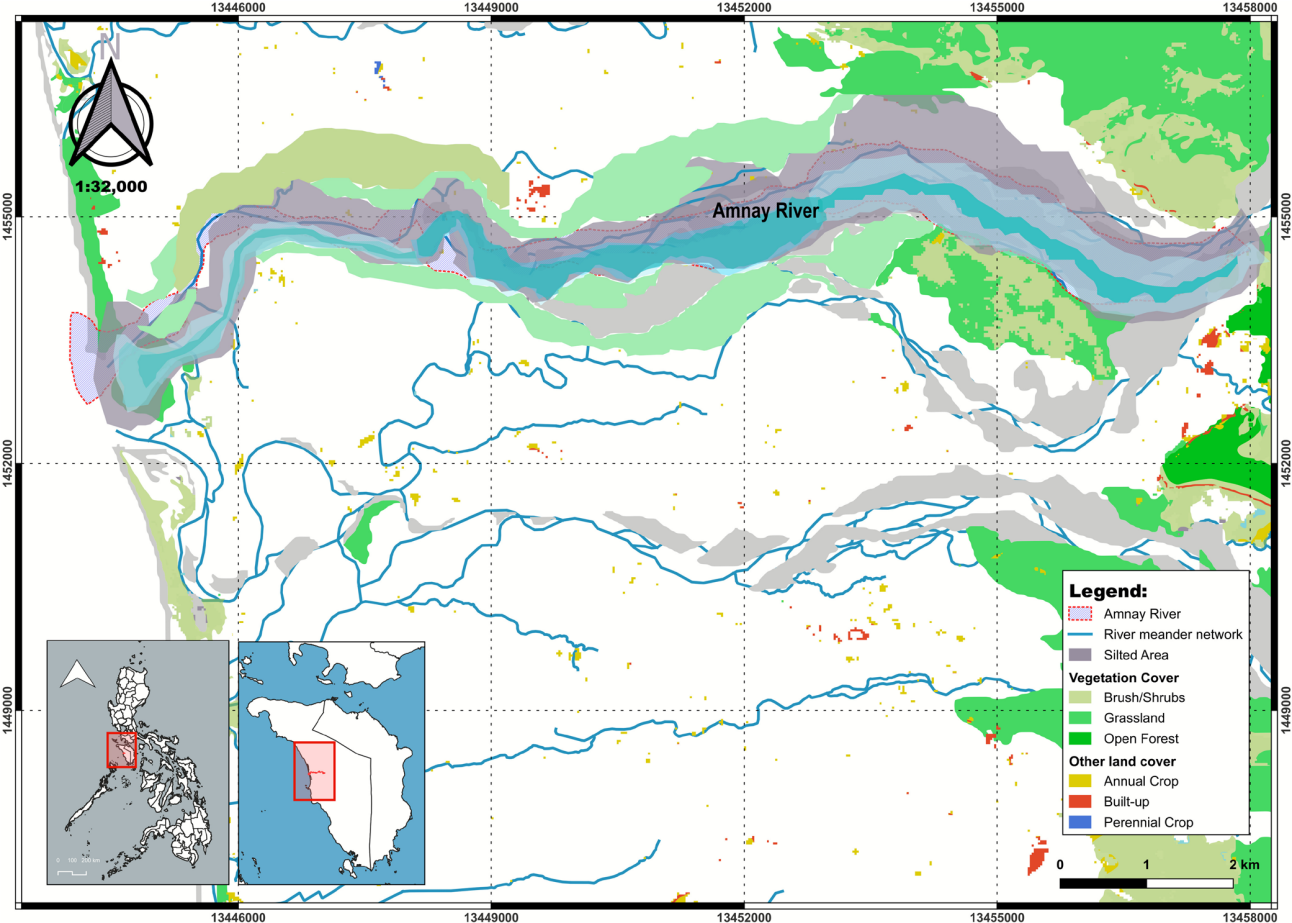
Different vegetation communities along the downstream section of Amnay River.

This study was carried along the downstream section of the Amnay River, extending to the river delta, with an emphasis on the riparian zones and associated vegetation. Located in Barangay Claudio Salgado, Sablayan, Occidental Mindoro, Philippines (12.944° N, 120.773° E) (Figure 1), the area is characterized by seasonally flooded riparian habitats and fragmented vegetation patches that are influenced by both natural and anthropogenic processes. There are three notable vegetation zones in the area: grasslands, shrubland having sparse vegetation, and an open forest (Figure 2). Topographically, the area is flat, crossed by the Amnay River. Generally, average annual rainfall is 2422 mm. According to the Modified Corona Climate Classification of PAGASA, Sablayan municipality falls under a Type I climate. This means that the area experiences two distinct seasons: a wet season that usually brings the heaviest precipitation from May to October, and a dry season that generally occurs from January to April (ARRDP‐EISR 2023).

The downstream section of the Amnay River is characterized by widespread and spatially variable sediment deposition, with no clearly defined or persistent low‐siltation reference sites. Consequently, the study focused on documenting existing vegetation conditions rather than conducting a quantitative comparison across siltation gradients.

### 2.2 Data Collection

**FIGURE 3:**
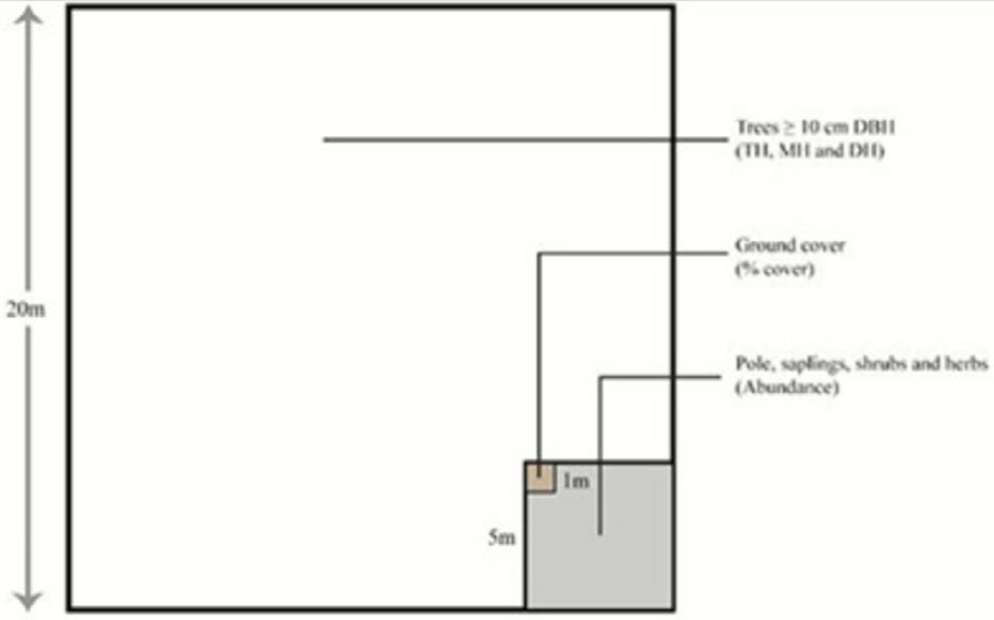
Modified quadrat‐based sampling design showing a 20 × 20 m main plot for trees (≥ 10 cm DBH) and nested sub‐quadrats for understorey vegetation and ground cover assessment.

Vegetation sampling was conducted along the downstream reaches of the Amnay River using a quadrat‐based approach. A total of nine (9) sampling sites were established along the river corridor, distributed longitudinally across the downstream section (Figure 3). Quadrat locations were purposively selected rather than systematically spaced, based on the presence of remnant riparian vegetation, substrate conditions, and accessibility within a highly sediment‐influenced and fragmented river environment. Although sampling sites were generally separated by a few hundred meters along the river course, they were not placed at fixed distance intervals. This design was intended to document existing riparian vegetation conditions rather than to impose uniform spacing in an area where vegetation occurs discontinuously due to widespread sediment deposition and channel instability. At each site, a single quadrat was placed within the riparian zone in areas where remnant vegetation was present. Quadrat placement was purposive rather than strictly systematic due to the fragmented and discontinuous nature of riparian vegetation. This design was intended to provide a baseline assessment of species composition rather than a comprehensive quantitative inventory. The survey technique employed followed the guidelines outlined by the DENR‐Biodiversity Management Bureau (BMB) Technical Bulletin No. 2016‐05 on flora diversity assessment (Malabrigo et al. 2018). Vegetation structure was classified in the field based on growth form and vertical stratification. All recorded plants were categorized as trees, shrubs, herbs, or grasses following ecological definitions by Grabherr and Kojima (1993) which involves vegetation units of similar physiognomy or appearance. Trees were defined as woody plants ≥ 3 m in height, shrubs as woody plants < 3 m, and herbs and grasses as non‐woody species. At the community level, vegetation units were further characterized based on the height and estimated cover of dominant species within sampling quadrats, following the vegetation categorization framework proposed by Loidi (2025) and a modified application of the structural vegetation classification of Specht (1970). This approach allowed the delineation of physiognomically distinct vegetation units along the riparian corridor. Species composition within each quadrat was determined through direct field identification using regional floras and field guides. When field identification was uncertain, specimens were photographed and later verified using taxonomic references. Species presence was recorded for each quadrat. Species were documented in situ and classified into native, secondary, and their eco‐class categories. Conservation status was verified using DENR (2017) Department Administrative Order (DAO) 2017–11 and the IUCN Red List Categories and Criteria (IUCN 2001) with its Red List ver.2025 3.1.

**FIGURE 4:**
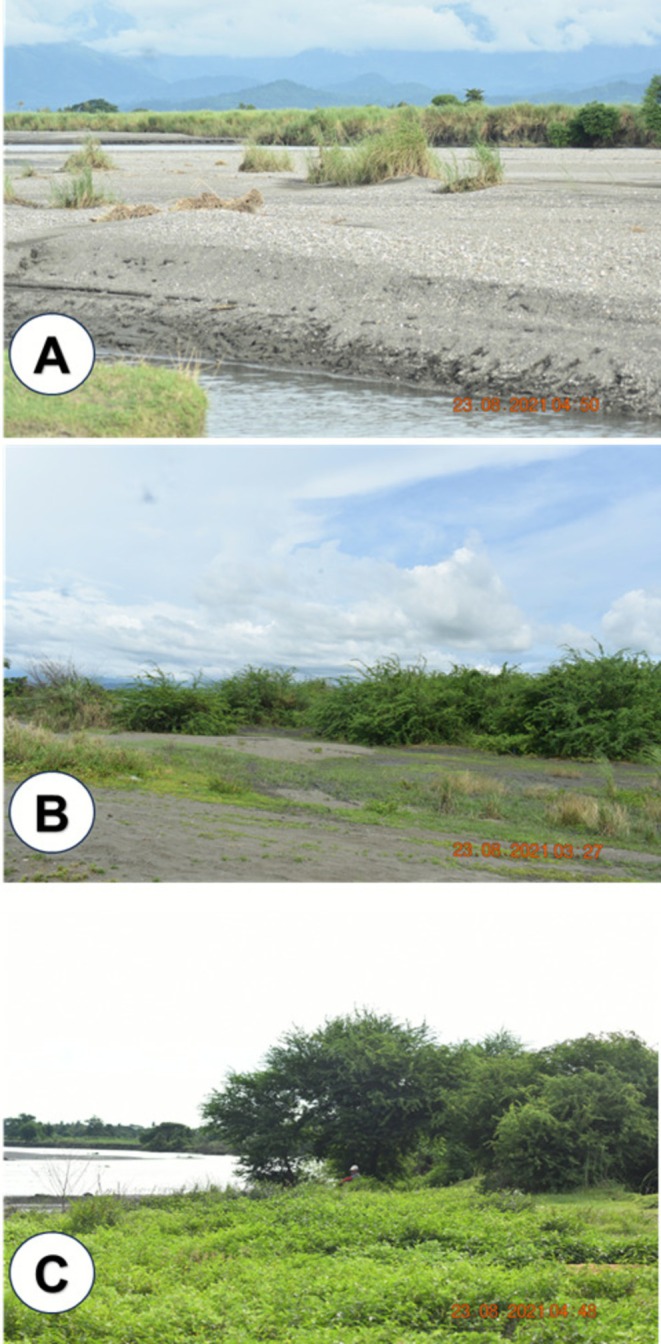
Representative habitat types in the study area: (A) sediment‐dominated open areas with exposed sand or silt and minimal vegetation cover; (B) herbaceous‐dominated riparian patches composed mainly of grasses and other non‐woody plants on shallow or periodically disturbed sediments; and (C) woody remnant vegetation patches consisting of shrubs and small trees persisting on relatively stable substrates.

Habitat types were identified in the field based on dominant vegetation structure, substrate condition, and degree of sediment deposition within the riparian zone (Figure 4). Given the highly disturbed and fragmented nature of the downstream reaches, habitat classification was qualitative and descriptive rather than quantitative. Three primary habitat types were recognized:

1. Sediment‐dominated open areas, characterized by exposed sand or silt substrates with little to no vegetation cover;
2. Herbaceous‐dominated riparian patches, consisting primarily of grasses and non‐woody plants established on shallow or periodically disturbed sediments; and
3. Woody remnant vegetation patches, composed of shrubs and small trees persisting on relatively more stable substrates.

Habitat differentiation was based on visual assessment of vegetation growth form, substrate stability, and evidence of recent sediment burial or erosion. These habitat types reflect current ecological conditions that reflect the remnant vegetation cover. Further, because sediment deposition was widespread and spatially heterogeneous throughout the downstream section, the study did not include a formal comparison between high‐ and low‐siltation areas. Instead, vegetation data were collected to characterize existing riparian conditions under the current sediment‐impacted environment.

### 2.3 Data Analysis

Richness of species and their relative abundance were summarized by habitat type. Information and biometrics gathered in the study area were encoded and characterized to describe flora compositions in the study area. The relative density (Rden), relative frequency (Rfreq), and relative dominance (Rdom) values for each plant species were evaluated to derive their species importance values (IVI), which is an ecological standard parameter in terrestrial flora ecology research that evaluates how ecologically dominant or significant a plant species (e.g., the ecological values and relationship) within a community and that provides information as to their species ranking, thereby reflecting their significance and contribution to the ecosystem. Species importance values were computed using the following formula following the assessment of Asigbaase et al. (2019):

$$\text{Density}=\frac{\text{number of individuals of}\;a\;\text{species}}{\text{total area of sampling quadrats}}$$

$$\text{Relative density}=\frac{\text{density of species}\;}{\text{density of}\;\mathrm{all}\;\text{species}}\times 100$$

$$\text{Frequency}=\frac{\text{number of quadrats the species were recorded}}{\text{total number of quadrats}}$$

$$\text{Relative frequency}=\frac{\text{frequency of}\;a\;\text{species}}{\text{total frequency of}\;\mathrm{all}\;\text{species}}\times 100$$

$$\text{Dominance}=\frac{\text{basal area of species}}{\text{total area of sampling quadrats}}$$

$$\text{Relative dominance}=\frac{\text{dominance of}\;a\;\text{species}\;}{\text{total dominance of}\;\mathrm{all}\;\text{species}\;}\times 100$$

$$\begin{array}{cc} \text{Importance value}\;\left(\mathrm{IV}\right)= & \text{Relative density}\;\left(\text{RDen}\right)\\ & +\text{relative dominance}\;\left(\text{RDom}\right)\\ & +\text{Relative frequency}\;\left(\text{RFreq}\right) \end{array}$$

Further, the diversity indices of all the quadrats were computed using widely used index calculations for species diversity such as Shannon, Simpson's, and Pielou Evenness index and referenced based on the occurrence and absence data for all the species found and recorded per quadrat. Given the limited number of sampling units, the absence of quantitative abundance data, and the highly fragmented nature of riparian vegetation, multivariate ordination or clustering analyses were not considered appropriate for this study.

## 3 Results

### 3.1 Spatial and Structural Characteristics of Riparian Vegetation

Riparian vegetation along the downstream reach of the Amnay River exhibited a discontinuous and spatially fragmented distribution. Vegetation occurred primarily as narrow linear strips along the riverbanks, interspersed with exposed substrates dominated by sand, gravel, and silt deposits. Three general vegetation physiognomic units were observed: (1) herbaceous‐dominated grasslands, (2) shrub‐dominated patches, and (3) isolated woody remnant stands.

Woody vegetation was unevenly distributed and largely restricted to small patches along relatively stable riverbanks. In contrast, extensive stretches of the riparian zone were characterized by open grassland and sparsely vegetated substrates. The mapped vegetation communities indicate limited lateral connectivity between patches, with several reaches lacking continuous canopy or understory cover.

### 3.2 Floristic Composition

A total of 125 vascular plant species, representing 106 genera and 40 families, were recorded across all sampling sites (Table 1). Angiosperms dominated the flora, with dicotyledonous species accounting for the majority of taxa. The most species‐rich families included Poaceae, Fabaceae, and Asteraceae, which collectively contributed a substantial proportion of the total species richness.

**TABLE 1:** Vascular flora orders, families, genera and species documented in the study area.

Order	Family	Genera	Species	Endemic	Species identified to genus level	Species identified to family level
Pteridophyta	0	0	0	0	0	0
Gymnospermae	0	0	0	0	0	0
*Angiospermae*						
Dicotyledonae	25	62	74	3	0	1
Monocotyledonae	21	44	51	0	0	0
Total	40	105	125	3	0	1

The floristic assemblage consisted of a mixture of native and non‐native species. Several taxa commonly associated with disturbed or open habitats were widely distributed across the study area. Species richness varied among sampling sites, with some transects supporting a limited number of species relative to others.

Dicotyledonae represents 25 families with 62 genera and 74 species, of which 52 noteworthy native species and three (3) Philippine Endemics were also identified. Monocotyledonae has 21 families with 44 genera, and 51 species have been identified. Common native riparian trees included *Terminalia catappa* , *Ficus nota* , and *Pterocarpus indicus* , while pioneer and disturbance‐associated species such as *Saccharum spontaneum* and *Ipomoea pes‐caprae* were abundant along eroded banks and sandbars. Bamboo ( *Bambusa blumeana* ) stands were observed stabilizing several riverbank sections, while agricultural encroachment contributed to the dominance of coconut ( *Cocos nucifera* ) and banana (*Musa* spp.) near settlements. Around 30 species from 12 families were recorded, of which at least 9 are small trees, 10 are medium‐size trees, and about 11 species are large trees. The most frequently observed and occurring plant species were Tan‐ag ( *Kleinhovia hospita* ), Bangkal (*Nauclea orientalis*), Acacia ( *Samanea saman* ), Aroma ( *Acacia farnesiana* ), Kakawate ( *Gliricidia sepium* ), Talisai (*Terminalia cattapa*), and Kamachile (*Pithecelobium dulce*). In plot 2, some mangrove species were only limited to Nipa ( *Nypa fruticans* ) and some individuals of Bakauan babae ( *Rhizophora mucronata* ). A relative coverage of the mixture is totaled by the density of stems per unit area (about 2–3 stems per 10 m^2^) having approximately five (5) Nipa ( *N. fruticans* ) stems and six (6) Bakauan babae ( *R. mucronata* ) stems in 100 m^2^.

### 3.3 Growth‐Form Composition and Vertical Structure

**FIGURE 5:**
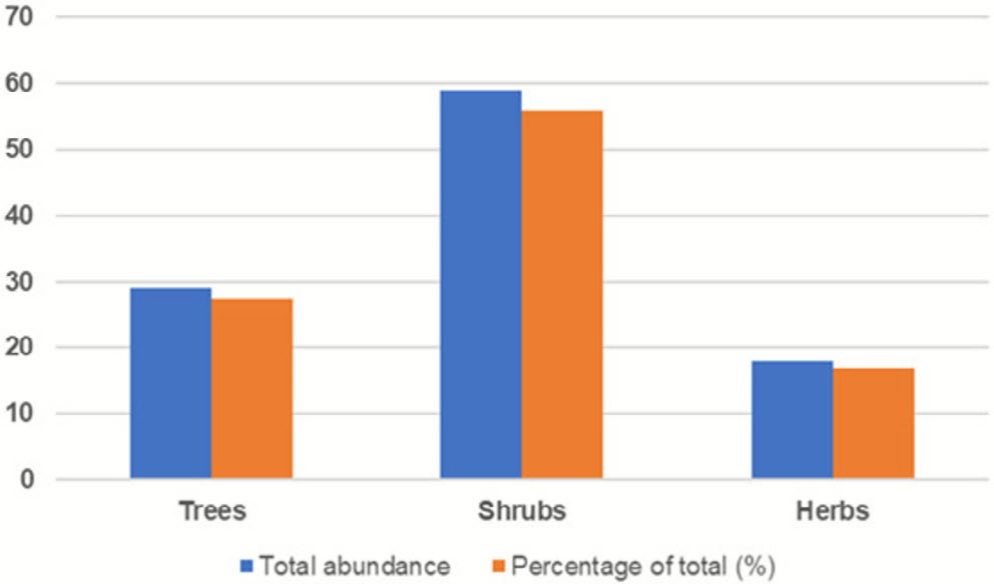
Relative contribution of major growth forms based on relative abundance of recorded individuals. Shrubs accounted for the largest proportion of vegetation (55.7%), followed by trees (27.4%) and herbaceous species (17.0%), indicating a dominance of woody growth forms within the study area.

The riparian vegetation exhibited clear differences in growth‐form composition, with shrubs contributing the largest proportion of individuals, followed by trees and herbaceous species (Figure 5).

#### 3.3.1 Tree Layer

The tree layer was characterized by low stem density and limited species richness. Tree individuals were primarily confined to remnant patches and were largely absent from actively aggrading or unstable substrates. Dominant tree species included *Nauclea orientalis* (L.) L., *Pithecelobium dulce* (Roxb) Benth., and *Samanea saman* (Jacq.) Merr., which exhibited the highest Importance Value Index (IVI) scores across sampling sites (Table 2).

**TABLE 2:** Top 10 trees with highest importance value (IV).

Scientific name	Common name	Family name	Count	RD	IV
*Nauclea orientalis* (L.) L.	Bankal	Rubiaceae	8	3.54	34.65
*Pithecelobium* dulce (Roxb) Benth.	Kamachile	Fabaceae	17	7.33	23.45
*Samanea saman* (Jacq.) Merr.	Acacia	Fabaceae	15	3.53	15.33
*Terminalia catappa* L.	Talisai	Combretaceae	14	6.03	14.33
*Trema orientalis* (L.) Blume	Anabiong	Cannabaceae	11	4.74	13.10
*Acacia farnesiana* (L.) Willd.	Aroma	Fabaceae	8	3.45	11.12
*Albizia procera* (Roxb.) Benth	Akleng Parang	Fabaceae	8	3.10	10.32
*Cordia dichotoma* G. Forster	Anonang	Boraginaceae	8	2.59	10.21
*Melanolepis multiglandulosa* (Reinw. Ex Blume) Reichb. f. & Zoll.	Alim	Euphorbiaceae	6	3.88	8.65
*Lagerstroemia speciosa* (L.) Pers.	Banaba	Lamiaceae	9	2.45	7.92

#### 3.3.2 Understorey Layer

The understorey layer consisted mainly of shrubs and juvenile trees, with species composition dominated by disturbance‐tolerant taxa. Understorey vegetation showed uneven distribution, with higher abundance near remnant woody stands and reduced presence in open, sediment‐dominated areas. Abundance of all the understorey species documented is presented in Table 3.

**TABLE 3:** Top 10 most abundant understorey species.

Scientific name	Common name	Family name	Abundance
*Nauclea orientalis* Lam.	Bangkal	Rubiaceae	20
*Lycianthes biflora* (Lour.) Bitter	Bagan bagan	Solanaceae	15
*Stachytarpheta jamaicensis* (L.) Vahl.	Bolomaros	Verbenaceae	11
*Tabernaemontana pandacaqui* Poir.	Pandakaki	Apocynaceae	10
*Lantana camara L*.	Koronitas	Lamiaceae	10
*Ficus ulmifolia* Lamk	Is‐Is	Moraceae	9
*Chromolaena odorata* (L.) R.M. King & H. Rob.	Hagonoi	Asteraceae	9
*Senna alata*	Akapulko	Fabaceae	8
*Melochia concatenata L*.	Marasaluyot	Malvaceae	7
*Homonoia riparia* Lour.	Lumanai	Euphorbiaceae	7

#### 3.3.3 Ground Cover

In terms of ground cover, the majority of the individuals are represented by the families of Lamiaceae, Fabaceae, Commelinaceae, Malvaceae, and Poaceae such as *Sida acuta* , *Crotalaria pallida* , *Hyptis suaveolens* , *Alysicarpus vaginalis, Alternanthera sessilis*, *Commelina benghalensis* , and *Urena lobata* . Others are leguminous and weedy ground cover species that are common to open areas and disturbed sections such as those from the open areas and grasslands, which include Makahiya ( *Mimosa pudica* ), Balatong aso ( *Calopogonium mucunoides* ), and Dagad ( *Tridax procumbens* ). Ground cover is also occasionally dense in exposed sections, usually in areas that form a small ravine and are shaded under rocks with dense tree vegetation.

Overall, forty‐one (41) ground cover species recorded from all 1 m x 1 m quadrats was recorded (Table 4). It must be noted that the ground cover species referred to in this study include species of different plant habits and forms such as crawling or erect inside the 1 m × 1 m quadrat and with a height of less than 1 m. Hence, wildlings or germinated seeds of different tree species are included as ground cover.

**TABLE 4:** Ten most dominant ground cover species and substrate recorded in the all‐sampling quadrats.

Scientific name	Common name	Family name	Relative % cover
Litter	—	—	5.7
Rocks	—	—	50.0
Bare	—	—	15.0
*Sida acuta L*.	Walis‐walisan	Malvaceae	1.5
*Crotalaria pallida* Aiton	Payang	Fabaceae	1.05
*Hyptis suaveolens* (L.) Poit.	Suob kabayo	Lamiaceae	1.00
*Imperata cylindrica* (L.) Beauv.	Cogon	Poaceae	0.1
*Alternanthera sessilis* (L.) R. Br. Ex DC.	Butonesan	Malvaceae	0.1
*Commelina benghalensis L*.	Alikbangon babae	Commelinaceae	0.05
*Calopogonium mucunoides Desv*.	Balatong aso	Fabaceae	0.05
*Dactyloctenium aegyptium* (L.) Willd.	Paang uwak	Poaceae	0.05
*Piper umbellatum L*.	Kubamba	Piperaceae	0.05
*Passiflora foetida L*.	Karunggut	Passifloraceae	0.02

### 3.4 Species Dominance and Ecological Importance

Species dominance patterns, as indicated by Importance Value Index calculations, revealed a small number of taxa contributing disproportionately to overall vegetation structure. Species with the highest IVI values were primarily fast‐growing, disturbance‐tolerant plants capable of colonizing open substrates. These species were consistently recorded across multiple transects, indicating broad spatial distribution within the study area.

**FIGURE 6:**
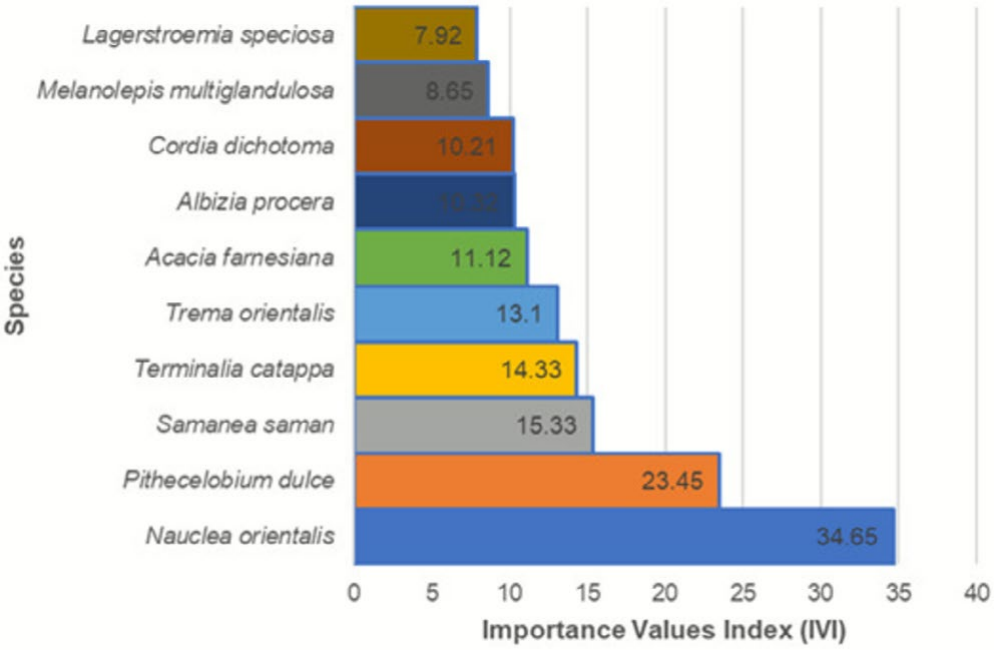
Species dominance patterns based on the Importance Value Index (IVI). Species are arranged from highest to lowest IVI, highlighting the most ecologically dominant species in the study area.

In contrast, species with lower IVI values were generally restricted to specific sites or microhabitats, often within remnant woody patches (Figure 6). Species dominance was strongly skewed, with a small number of taxa exhibiting disproportionately high IVI values.

### 3.5 Conservation Status and Noteworthy Species

**FIGURE 7:**
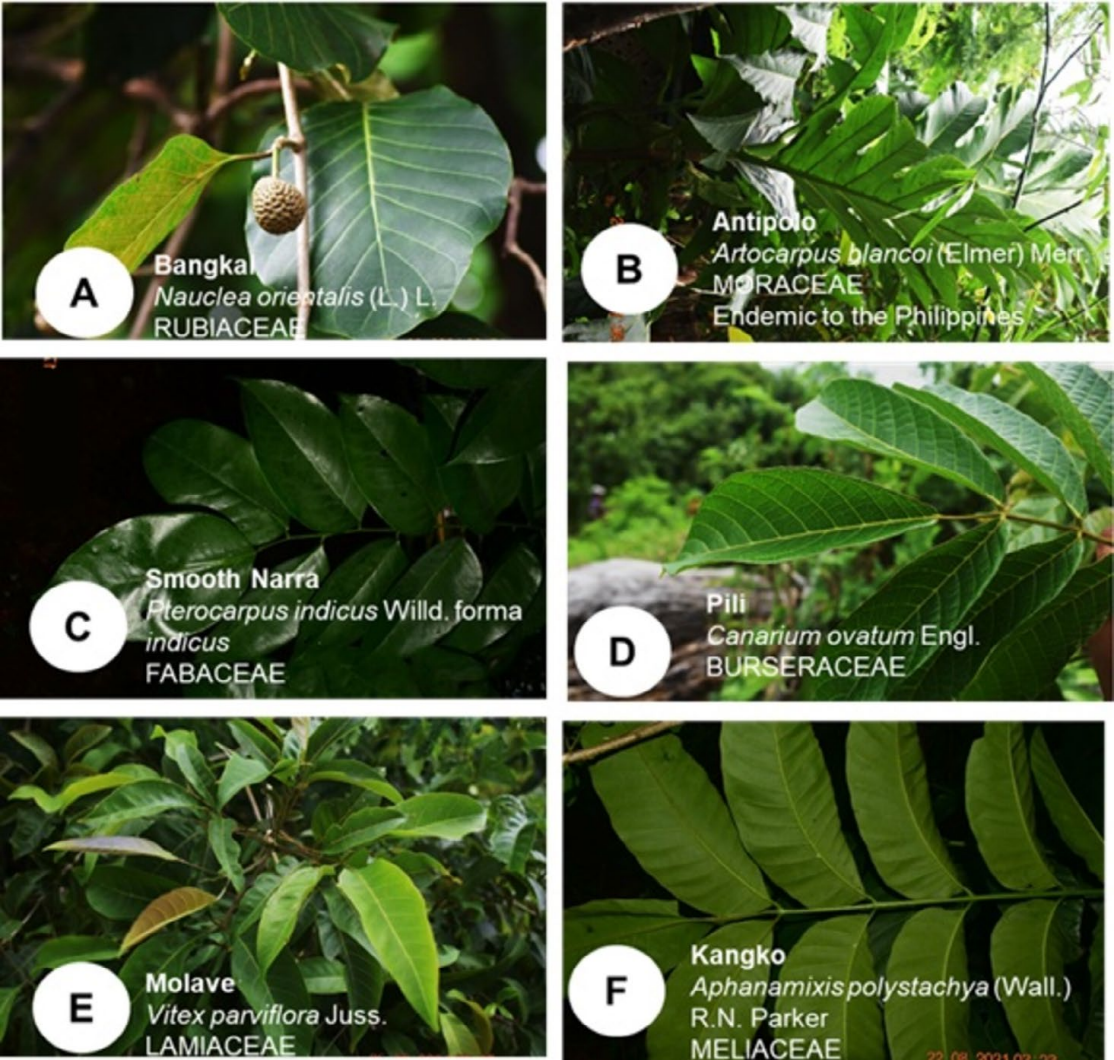
Photos of some documented indigenous tree species in the riparian banks of Amnay River.

Several native and locally significant species were recorded during the survey. A subset of taxa identified in the study area are recognized under national or international conservation frameworks, including species classified as endemic or of conservation concern. These species were typically confined to remnant vegetation patches and were absent from highly disturbed or sparsely vegetated areas. Photographic documentation of selected indigenous and noteworthy species is presented in Figure 7.

### 3.6 Patterns of Species Diversity Across Sampling Sites

Species diversity indices varied among sampling sites. Shannon–Wiener diversity values ranged from 1.795 to 2.676, while Simpson's diversity index and Pielou's evenness also showed spatial variation (Table 5). Sites with relatively higher diversity generally supported a greater mix of growth forms, whereas sites dominated by herbaceous cover exhibited lower diversity and evenness.

**TABLE 5:** Diversity indices of each quadrat and number of species recorded.

Plot	Diversity indices			No. of plant species	No. of individuals
	Shannon index (*H*′)	Simpson index (*D*′)	Evenness (*J*)		
1	2.449	0.843	0.761	25	133
2	2.287	0.886	0.825	17	83
3	2.044	0.814	0.797	23	105
4	1.541	0.610	0.556	19	106
5	1.989	0.843	0.801	15	107
6	1.951	0.819	0.785	11	95
7	1.795	0.819	0.780	19	101
8	2.676	0.792	0.806	14	111
9	2.013	0.840	0.785	24	105

No distinct clustering of sites based on diversity indices alone was observed, reflecting heterogeneity in local vegetation conditions along the river corridor.

### 3.7 Summary of Riparian Vegetation Condition

Overall, riparian vegetation along the downstream Amnay River is characterized by fragmented spatial distribution, low structural complexity, and dominance of disturbance‐tolerant species. Woody vegetation persists mainly as isolated remnant patches, while extensive portions of the riparian zone are occupied by herbaceous ground cover or remain sparsely vegetated.

## 4 Discussion

### 4.1 Riparian Vegetation Structure in a Sediment‐Influenced River System

The fragmented and discontinuous riparian vegetation structure documented along the downstream Amnay River reflects conditions typical of river corridors influenced by frequent sediment deposition and substrate instability. In fluvial systems, repeated burial of seedlings and scouring during high‐flow events limit the establishment of woody vegetation and favor early‐successional growth forms (Clements 1997; Naiman et al. 2005; Corenblit et al. 2007). This pattern is evident in the present study, where extensive portions of the riparian zone are dominated by herbaceous vegetation, while trees are largely confined to isolated remnant patches. Similar vegetation configurations have been reported in tropical and subtropical rivers where geomorphic processes strongly regulate riparian plant recruitment and persistence (Tabacchi et al. 1998; Gurnell et al. 2016). The spatial restriction of woody vegetation in the Amnay River suggests that suitable microsites for tree establishment are limited, likely occurring only where bank stability and substrate cohesion are sufficient to support long‐term growth.

### 4.2 Floristic Composition and Dominance of Disturbance‐Tolerant Taxa

The floristic composition of the riparian zone, characterized by the dominance of Poaceae, Fabaceae, and other disturbance‐adapted families, is consistent with vegetation assemblages commonly observed in disturbed riparian environments (Replan et al. 2023). Species belonging to these families often exhibit rapid growth, high reproductive output, and tolerance to fluctuating moisture and substrate conditions, enabling them to colonize and persist in open, sediment‐rich habitats (Corenblit et al. 2009). The prevalence of such taxa in the Amnay River aligns with findings from other Philippine and Southeast Asian river systems (Mitsui and Setoguchi 2012; Malabrigo Jr. et al. 2020), where land‐use change and sediment influx promote the expansion of herbaceous and shrub species at the expense of late‐successional woody vegetation (Lasco et al. 2001; Garcia et al. 2014). These floristic patterns indicate a vegetation assemblage shaped largely by physical disturbance rather than competitive exclusion.

### 4.3 Structural Simplicity and Reduced Vertical Complexity

The limited vertical stratification observed in the study area—particularly the low density of mature trees and sparse understorey development—reflects a structurally simplified riparian corridor. Vertical complexity is a key attribute of intact riparian systems, contributing to habitat diversity and ecological stability (Naiman and Décamps 1997). In contrast, structurally simplified riparian zones are commonly associated with frequent disturbance and reduced successional progression. Reduced plant diversity and simplified vegetation structure may also affect belowground processes and ecosystem functioning, as plant diversity has been shown to influence soil microbial biomass and nutrient dynamics (Eisenhauer et al. 2017).

In the Amnay River, the dominance of ground‐layer vegetation and the scarcity of multi‐layered canopy structure suggest constrained successional development. Such conditions have been linked to sediment aggradation, ecosystem productivity and bank instability in tropical river systems, which disrupt the establishment and persistence of woody vegetation (Gurnell et al. 2016; Lange et al. 2020). Shrub‐dominated vegetation observed along the Amnay River may also be interpreted as a successional stage following the removal or loss of mature riparian forest cover. Xian et al. (2015) described shrubland vegetation as woody successional communities that regenerate after forest disturbance, a condition commonly observed in riparian environments subject to repeated physical or anthropogenic disturbance. Riparian vegetation in such settings is often characterized by a mixed composition of trees, shrubs, and tall herbaceous species, reflecting ongoing successional processes and inherent structural instability. The prevalence of these vegetation units in the study area underscores the dynamic nature of riparian vegetation and highlights the importance of baseline documentation for future monitoring of successional trajectories.

### 4.4 Spatial Variation in Species Diversity Along the River Corridor

Variation in species diversity indices among sampling sites likely reflects localized differences in habitat conditions, including substrate stability, vegetation cover, and proximity to remnant woody patches. Sites exhibiting relatively higher diversity tended to support a mixture of growth forms, whereas sites dominated by herbaceous cover showed lower diversity and evenness. Comparable studies have shown that riparian species diversity often declines in fragmented and disturbed landscapes, where habitat heterogeneity and connectivity are reduced (Nilsson and Svedmark 2002; Malabrigo Jr. et al. 2022). The absence of clear diversity gradients along the Amnay River highlights the heterogeneous nature of disturbance and recovery processes operating at fine spatial scales within the riparian corridor.

### 4.5 Ecological Significance of Remnant Riparian Vegetation

Although limited in extent, remnant woody vegetation patches identified in this study may play an important ecological role in the riparian landscape. Such patches can function as refuge for native species (Coelho et al. 2020) and serve as potential sources of propagules for natural regeneration (Naiman et al. 2005). Several native tree species recorded within these remnants are known to contribute to bank stabilization and microhabitat formation, which are critical functions in riparian ecosystems.

The persistence of these remnant stands suggests that, despite widespread disturbance, elements of the original riparian vegetation remain and may be important focal points for conservation and management initiatives.

### 4.6 Study Limitations and Value as a Baseline Assessment

This study represents a baseline assessment of riparian vegetation along the downstream Amnay River and is subject to several limitations. The absence of relatively undisturbed reference sites restricts direct comparison across disturbance gradients, and the study relies primarily on floristic composition and structural attributes rather than long‐term monitoring or experimental manipulation. Nevertheless, baseline floristic and structural data are essential for understanding current vegetation conditions (Pollock et al. 2012) and for tracking future changes in riparian systems (Kent 2012). The results presented here provide a foundation for subsequent comparative studies, long‐term monitoring, and the evaluation of riparian restoration and management efforts in sediment‐influenced tropical river systems.

## 5 Conclusion

This study provides a baseline assessment of the floristic composition and structural characteristics of riparian vegetation along the downstream reach of the Amnay River. The riparian corridor is characterized by a fragmented spatial pattern, limited vertical complexity, and a vegetation assemblage dominated by herbaceous and disturbance‐tolerant species. Woody vegetation persists mainly as isolated remnant patches, highlighting the constrained availability of stable substrates capable of supporting long‐term tree establishment. The observed floristic patterns reflect the strong influence of physical disturbance and sediment dynamics on riparian vegetation structure. Variation in species diversity among sampling sites underscores the heterogeneous nature of riparian habitats within the river corridor, shaped by localized differences in substrate conditions and vegetation cover rather than a uniform disturbance gradient. Despite its limitations, particularly the absence of relatively undisturbed reference sites, this study establishes an important baseline for understanding current riparian vegetation conditions in a sediment‐influenced tropical river system. The documentation of remnant woody vegetation and native species provides a foundation for future monitoring and comparative studies, as well as for evaluating changes in riparian structure over time. Overall, the findings contribute to the growing body of literature on tropical riparian vegetation dynamics and emphasize the value of baseline floristic assessments in river systems experiencing ongoing physical disturbance. Future research incorporating long‐term monitoring and broader spatial comparisons would further improve understanding of riparian vegetation responses to hydrological and geomorphic processes.

## Funding

The author has nothing to report.

## Conflicts of Interest

The author declares no conflicts of interest.

## Data Availability

This dataset contains the vegetation analysis data for vascular plant taxa recorded along the downstream section of the Amnay River, Sablayan, Occidental Mindoro, Philippines. The data supporting the findings of this study are openly available in Zenodo at https://doi.org/10.5281/zenodo.18321869. The dataset includes plot‐level diversity indices, species richness, and total abundance values corresponding to the tables presented in the manuscript.
